# Supplementary material for: Translatome analysis reveals altered serine and glycine metabolism in T-cell acute lymphoblastic leukemia cells
Source: Nat Commun. 2019 Jun 11;10:2542. doi: 10.1038/s41467-019-10508-2 (PMC6559966; doi:10.1038/s41467-019-10508-2)
Supplement: Supplementary file 1 — Supplementary Information [file 41467_2019_10508_MOESM1_ESM.pdf]

SUPPLEMENTARY INFORMATION

Translatome analysis reveals altered serine and glycine metabolism in T-cell acute lymphoblastic leukemia cells

Kim R. Kampen[1]†, Laura Fancello[1]†, Tiziana Girardi[1], Gianmarco Rinaldi[2,3], Mélanie Planque[2,3], Sergey O. Sulima[1], Fabricio Loayza-Puch[4], Benno Verbelen[1], Stijn Vereecke[1], Jelle Verbeeck[1], Joyce Op de Beeck[1], Jonathan Royaert[1], Pieter Vermeersch[5], David Cassiman[6], Jan Cools[7,8] , Reuven Agami[9,10], Mark Fiers[11], Sarah-Maria Fendt[2,3], Kim De Keersmaecker[1]

1 Laboratory for Disease Mechanisms in Cancer, Department of Oncology, KU Leuven - University of Leuven, LKI - Leuven Cancer Institute, Herestraat 49, 3000, Leuven, Belgium
2 Laboratory of Cellular Metabolism and Metabolic Regulation, VIB-KU Leuven Center for Cancer Biology, VIB, Herestraat 49, 3000 Leuven, Belgium
3 Laboratory of Cellular Metabolism and Metabolic Regulation, Department of Oncology, KU Leuven and Leuven Cancer Institute (LKI), Herestraat 49, 3000 Leuven, Belgium
4 Translational Control and Metabolism, German Cancer Research Center (DKFZ), Heidelberg, Germany
5 Department of Laboratory Medicine, University Hospitals Leuven, Herestraat 49, 3000, Leuven, Belgium
6 Department of Gastroenterology-Hepatology and Metabolic Center, University Hospitals Leuven, Herestraat 49, 3000, Leuven, Belgium
7 Laboratory of Molecular Biology of Leukemia, VIB-KU Leuven Center for Cancer Biology, VIB, Herestraat 49, 3000 Leuven, Belgium
8 Laboratory of Molecular Biology of Leukemia, Center for Human Genetics, KU Leuven and Leuven Cancer Institute (LKI), Herestraat 49, 3000 Leuven, Belgium
9 Department of Pediatric Oncology/Hematology, Erasmus Medical Center, Wytemaweg 80, 3015 CN, Rotterdam, the Netherlands
10 Division of Oncogenomics, The Netherlands Cancer Institute, Plesmanlaan 121, 1066 CX, Amsterdam, The Netherlands
11 Laboratory for the Research of Neurodegenerative Diseases, VIB-KU Leuven Center for Brain & Disease Research, Herestraat 49, 3000 Leuven, Belgium

† These authors contributed equally to this work

A

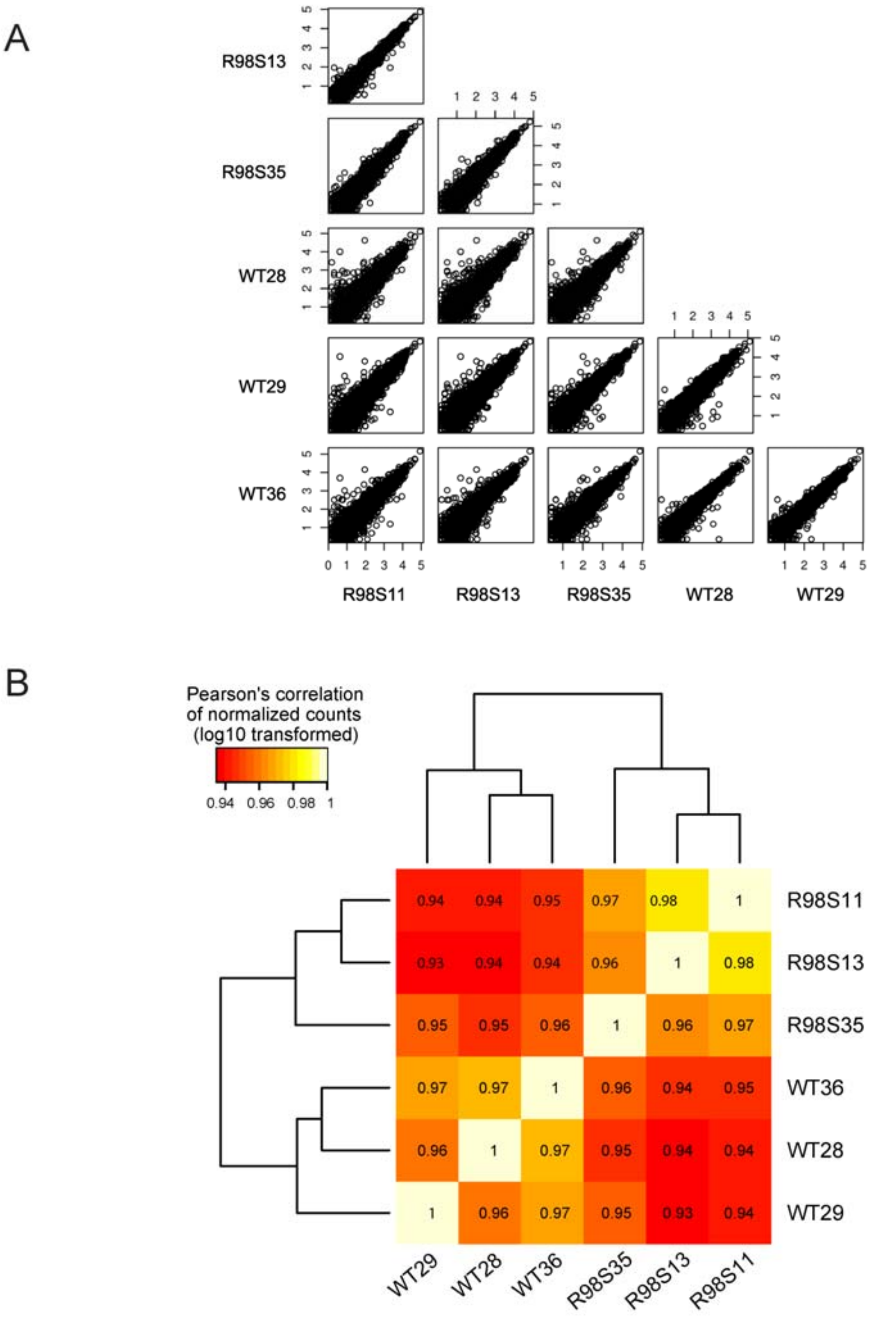

B

Supplementary Figure 1. Ribosome footprinting results are highly reproducible across biological replicates.

A. Correlation between biological ribosome footprinting replicates (log10-transformed normalized ribosome footprint counts). **B.** Hierarchical clustering of Pearson correlation coefficients between biological ribosome footprinting replicates (calculated on log10-transformed normalized ribosome footprint counts).

A

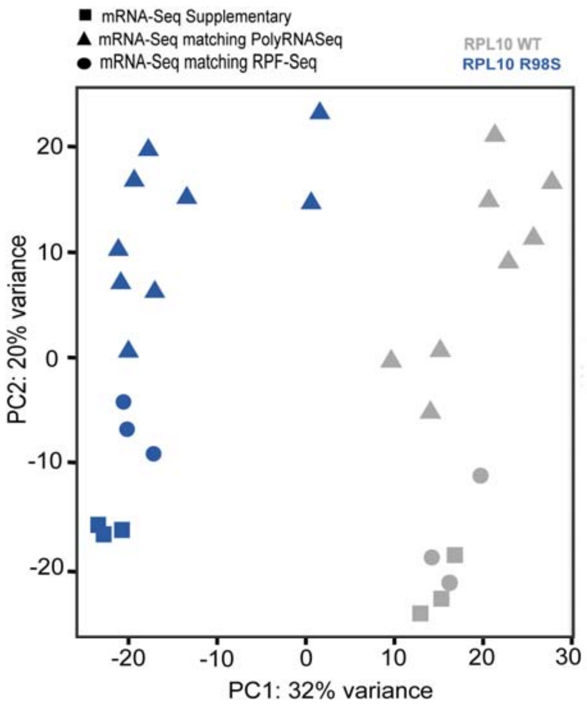

B

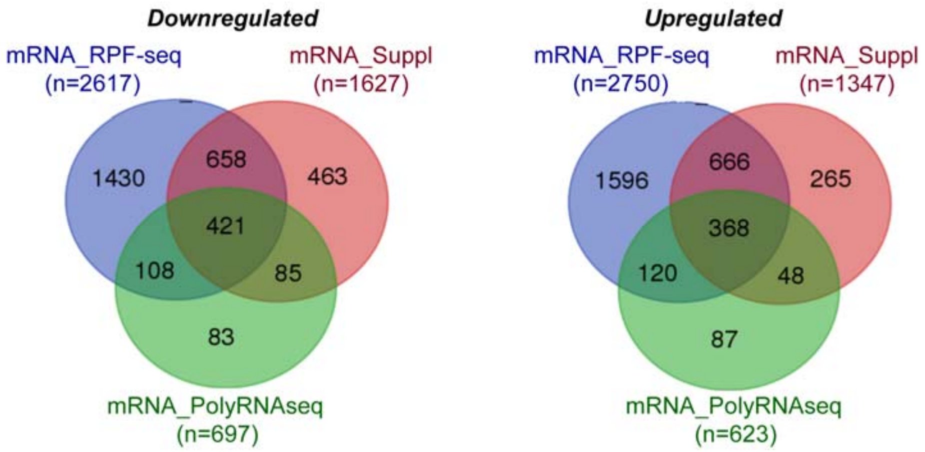

Supplementary Figure 2. Analysis of three total mRNA sequencing datasets identifies consistent transcriptional changes between *RPL10 WT* and *R98S* cells

A. Principal component analysis based on total mRNA levels (DESeq2 normalized read counts). Three different total mRNA sequencing datasets are represented: the dataset matching the ribosome footprinting, the one matching polysomal RNA sequencing and a third independent one. **B.** Venn diagrams showing the overlap of differentially expressed genes (DESeq2 FDR<0.1, two-sided Wald test with Benjamini-Hochberg correction, n=3 biologically independent *RPL10 WT* and *R98S* Ba/F3 clones) identified in each mRNA sequencing dataset. mRNA_PolyRNAseq: mRNA dataset matching polysomal RNA sequencing; mRNA_RPF-seq: mRNA dataset matching ribosome footprinting; mRNA_Suppl: additional mRNA dataset.

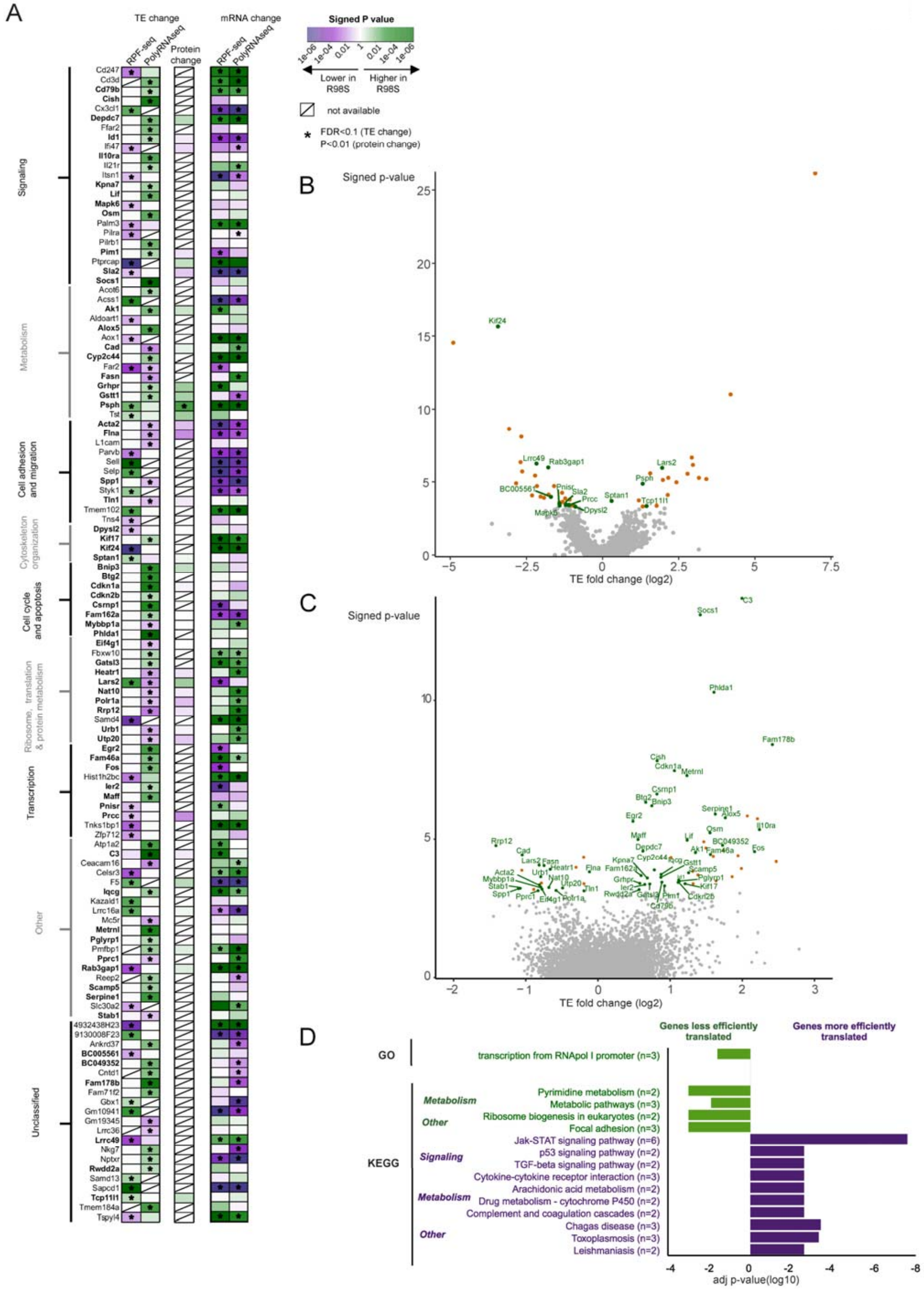
A
TE change
mRNA change
RPF-seq
PolyRNAseq
Protein change
Signed P value
Lower in R98S
Higher in R98S
not available
FDR<0.1 (TE change)
P<0.01 (protein change)
Signaling
Metabolism
Cell adhesion and migration
Cytoskeleton organization
Cell cycle and apoptosis
Ribosome, translation & protein metabolism
Transcription
Other
Unclassified
B
Signed p-value
TE fold change (log2)
C
Signed p-value
TE fold change (log2)
D
Genes less efficiently translated
Genes more efficiently translated
GO
transcription from RNApol I promoter (n=3)
KEGG
Metabolism
Other
Pyrimidine metabolism (n=2)
Metabolic pathways (n=3)
Ribosome biogenesis in eukaryotes (n=2)
Focal adhesion (n=3)
Signaling
Jak-STAT signaling pathway (n=6)
p53 signaling pathway (n=2)
TGF-beta signaling pathway (n=2)
Cytokine-cytokine receptor interaction (n=3)
Metabolism
Arachidonic acid metabolism (n=2)
Drug metabolism - cytochrome P450 (n=2)
Complement and coagulation cascades (n=2)
Other
Chagas disease (n=3)
Toxoplasmosis (n=3)
Leishmaniasis (n=2)
adj p-value(log10)

Supplementary Figure 3. Genes with significant differences in translational efficiency (TE) between *RPL10 R98S* and *RPL10 WT* cells.

A. Heatmap representing all protein-coding genes with significant changes in TE (Babel, Z-test with Benjamini-Hochberg correction, FDR<0.1, n=3 biologically independent *RPL10 WT* and *R98S* Ba/F3 clones), identified in ribosome footprinting (RPF-seq) or polysomal RNA sequencing (PolyRNAseq). For each of these genes, the corresponding protein level change (Protein change, quantitative mass spectrometry) is reported. Changes in total mRNA (mRNA change, DESeq2), according to the total mRNA dataset matching ribosome footprinting (RPF-seq) or polysomal RNA sequencing (PolyRNAseq) are also shown. The color scale represents the signed p-value for the significance of the change. Statistically significant changes are indicated by a star (*) and correspond to FDR<0.1 for TE changes (Babel, Z-test with Benjamini-Hochberg correction, n=3 biologically independent *RPL10 WT* and *R98S* Ba/F3 clones) and for mRNA changes (DESeq2, two-sided Wald test with Benjamini-Hochberg correction, n=3 biologically independent *RPL10 WT* and *R98S* Ba/F3 clones) and p-value<0.01 for protein changes (T-test on normalized spectra from quantitative mass spectrometry, n=3 biologically independent *RPL10 WT* and *R98S* Ba/F3 clones). Genes discarded in Babel analysis because of insufficient (<10) mRNA reads to calculate TE or genes with no corresponding protein measurement are indicated as not available. The 67 genes with higher sequencing coverage (more than 10 reads per sample in ribosome footprinting and/or polysomal RNA sequencing and in their matching mRNA) which are retained for further analyses are indicated in bold. **B.-C.** Volcano plots displaying differences in TE between *RPL10 R98S* and *RPL10 WT* in ribosome footprinting (B) or polysomal RNA sequencing (C). Orange dots: genes with a significant difference in TE (Babel, Z-test with Benjamini-Hochberg correction, FDR<0.1, n=3 biologically independent *RPL10 WT* and *R98S* Ba/F3 clones); Green dots: genes with a significant TE difference (Babel, Z-test with Benjamini-Hochberg correction, FDR<0.1, n=3 biologically independent *RPL10 WT* and *R98S* Ba/F3 clones) and higher sequencing coverage (more than 10 reads per sample in ribosome footprinting and/or polysomal RNA sequencing and in their matching total mRNA dataset) which are retained for further analyses. **D.** Significant enrichment (adjusted p-value<0.1, hypergeometric test with Benjamini-Hochberg correction) in Gene Ontology (GO) biological process categories or KEGG pathways (KEGG) for the restricted subset of 67 genes presenting differential TE in *RPL10 R98S* and higher sequencing coverage. Adjusted p-values were calculated by hypergeometric test using the WebGestalt online platform[1] and reported as log10-transformed. n: number of genes differentially expressed.

A

Model	mRNA (RPF-Seq)	TE (RPF-Seq)	mRNA (PolyRNASeq)	TE (PolyRNASeq)	AIC	Adjusted R-squared
Prot FC ~ mRNA FC	✓				521.02	0.344
Prot FC ~ mRNA FC + TE FC	✓	✓			515.12	0.363
Prot FC ~ mRNA FC			✓		526.61	0.328
Prot FC ~ mRNA FC + TE FC			✓	✓	505.52	0.389
Prot FC ~ mRNA FC + TE FC + mRNA FC + TE FC	✓	✓	✓	✓	502.19	0.403

B Prot FC ~ 0.29 + 0.98 mRNA FC

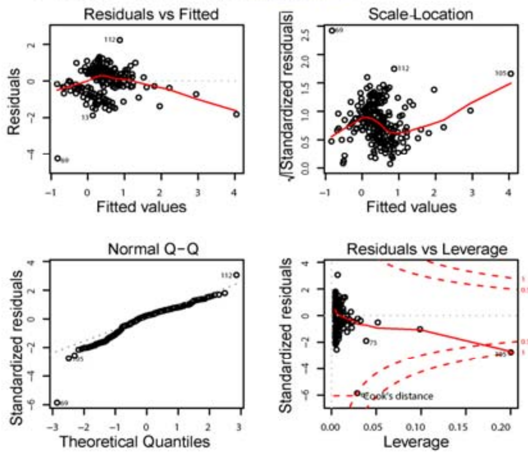

C Prot FC ~ 0.29 + 0.99 mRNA FC + 0.44 TE FC

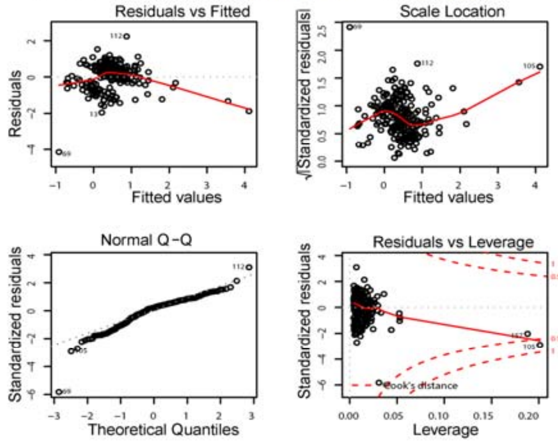

D Prot FC ~ 0.29 + 0.85 mRNA FC

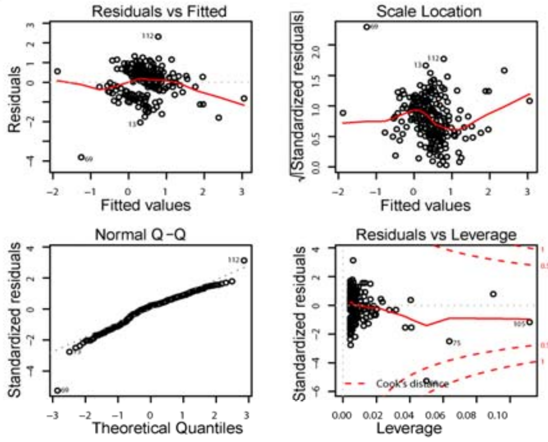

E Prot FC ~ 0.33 + 0.84 mRNA FC + 0.77 TE FC

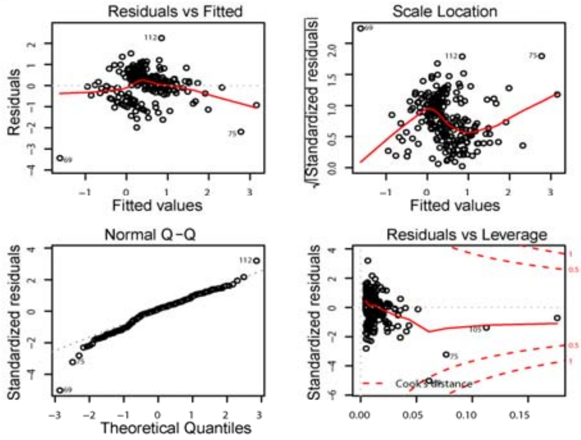

F Prot FC ~ 0.33 + 0.41 mRNA FC + 0.52 mRNA FC + 0.3 TE FC + 0.6 TE FC

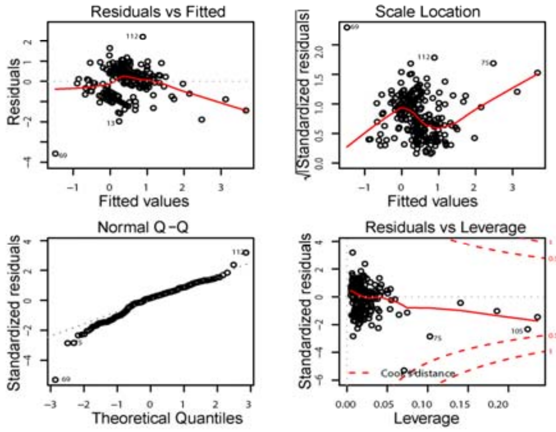

Supplementary Figure 4. Linear regression models based on mRNA and TE changes from combined ribosome footprinting and polysomal RNA sequencing better predict significant protein changes.

A. Comparison of linear regression models. Each model is defined by a linear equation and the variables used as predictors are indicated by ✓. The Akaike's Information Criterion (AIC) is a measure of the quality of a model based on the deviance and penalizing complex models which include irrelevant predictors. The lower the AIC score, the better the model. The coefficient of determination (adjusted R^2) is also reported as a measure of the proportion of total variation of significant protein changes that is explained by regression. **B.-F.** Diagnostic graphs for each linear regression model described by the linear equation on top. The blue color indicates predictors originating from ribosome footprinting and/or its matching mRNA dataset. The green color indicates predictors originating from polysomal RNA sequencing and/or its matching mRNA dataset. Diagnostic plots show that residual errors are random and normally distributed and that no outliers heavily affect the results, indicating good quality of the regression. Indeed, the residual errors (upper left) or square root of the standardized residuals (upper right) plotted versus their fitted values are randomly distributed around the horizontal line, with no obvious trend. The standard Q-Q plot (bottom left) shows that residual errors are normally distributed. Finally, the bottom right plot reports for each point its importance in determining the regression result (leverage). Cook's distance is also reported: distances larger than 1 may suggest the presence of an outlier).

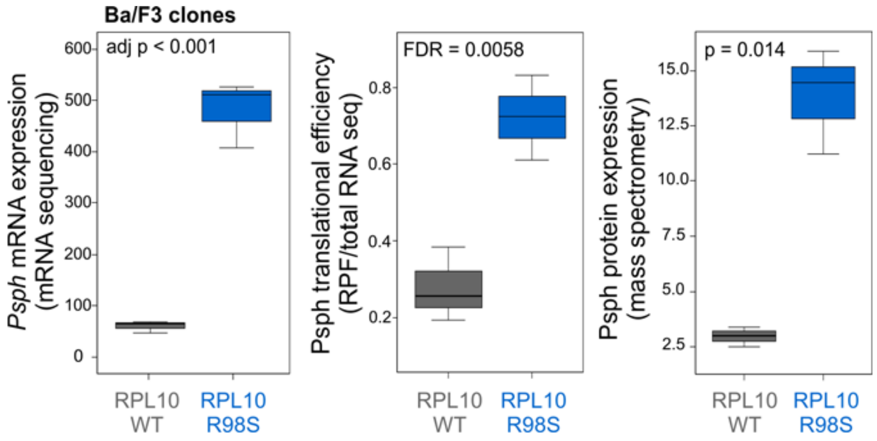

Supplementary Figure 5. Psph transcriptional and translational regulation in Ba/F3 *RPL10 WT* and *R98S* cells.

Left: *Psph* mRNA expression levels as measured by total mRNA sequencing. Middle: Psph translational efficiency based on ribosome footprinting counts and normalized for total mRNA levels. Right: Psph protein expression levels detected by quantitative mass spectrometry. Box-plots show the median and error bars define data distribution of three *RPL10 WT* (grey) clones and three *R98S* (blue) Ba/F3 clones. A two-sided Wald test with Benjamini-Hochberg correction was used for differences in mRNA expression levels, a Z-test with Benjamini-Hochberg correction for difference in TE and a T-test for differences in protein expression levels (n=3 biologically independent Ba/F3 clones for *RPL10 WT* and *R98S* conditions).

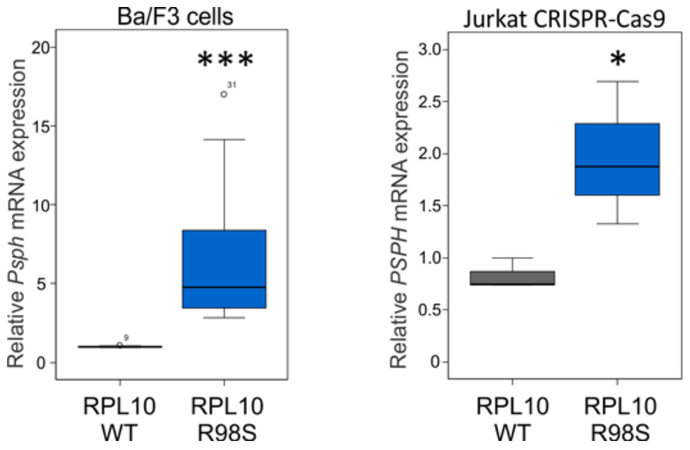

Supplementary Figure 6. qRT-PCR analysis of *Psph* mRNA expression levels in *RPL10 WT* and *R98S* cell models.

Psph mRNA expression levels in three *RPL10 WT* and three *R98S* Ba/F3 clones (left) and in CRISPR-Cas9 engineered Jurkat *RPL10 WT* and *R98S* cells. Box-plots show the median and error bars define data distribution. * p-value < 0.05; *** p-value < 0.001. P-values were calculated using a two-tailed student's t-test.

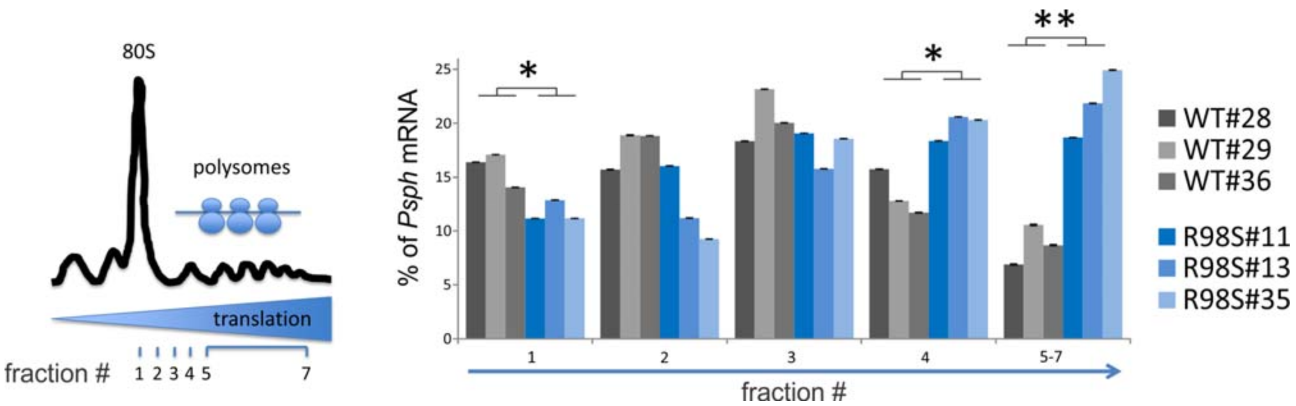

Supplementary Figure 7. Psph translational upregulation in Ba/F3 *RPL10 R98S* cells.

Left: Representative Ba/F3 polysome profile with indication of the fractions that were analyzed by qRT-PCR. Cell lysates from 3 *RPL10 WT* Ba/F3 cell clones and 3 *RPL10 R98S* Ba/F3 cell clones were applied on a sucrose gradient and fractionated into 7 fractions as indicated. For technical reasons, the three fractions corresponding to the heaviest polysomes (fractions 5-7) were combined and considered as 1 fraction. RNA was extracted from equal volumes of the 5 remaining fractions, reverse transcribed, and qRT-PCR was performed for the *Psph* mRNA. Right: Distribution of the *Psph* mRNA over the different fractions as assessed by qRT-PCR in three technical repeats. The % of *Psph* mRNA in the analyzed fraction is shown, with the *Psph* mRNA in all fractions of that sample together being 100%. The plot illustrates a shift in the distribution of ribosome bound *Psph* mRNA towards the most actively translating polysomal fractions in *RPL10 R98S* (blue bars) cells as compared to *WT* (grey bars) cells. Data are presented as mean ± SD. Statistical analysis * p-value < 0.05, ** p-value < 0.01. P-values were calculated using a two-tailed student's t-test.

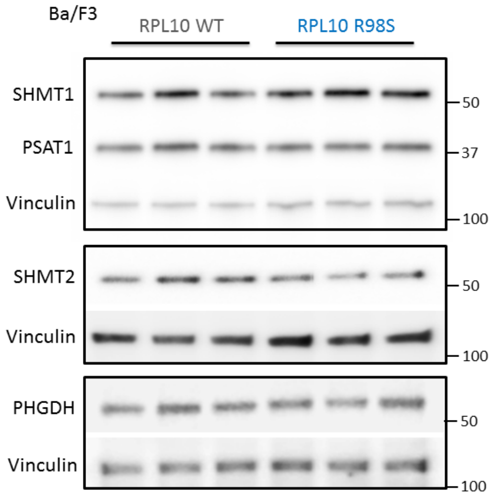

Supplementary Figure 8. Protein expression of other serine/glycine synthesis enzymes besides PSPH

PHGDH, PSAT1, SHMT1, and SHMT2 protein expression levels in *RPL10 WT* (grey) and *R98S* (blue) Ba/F3 clones as detected by immunoblot analysis (n=3 versus n=3).

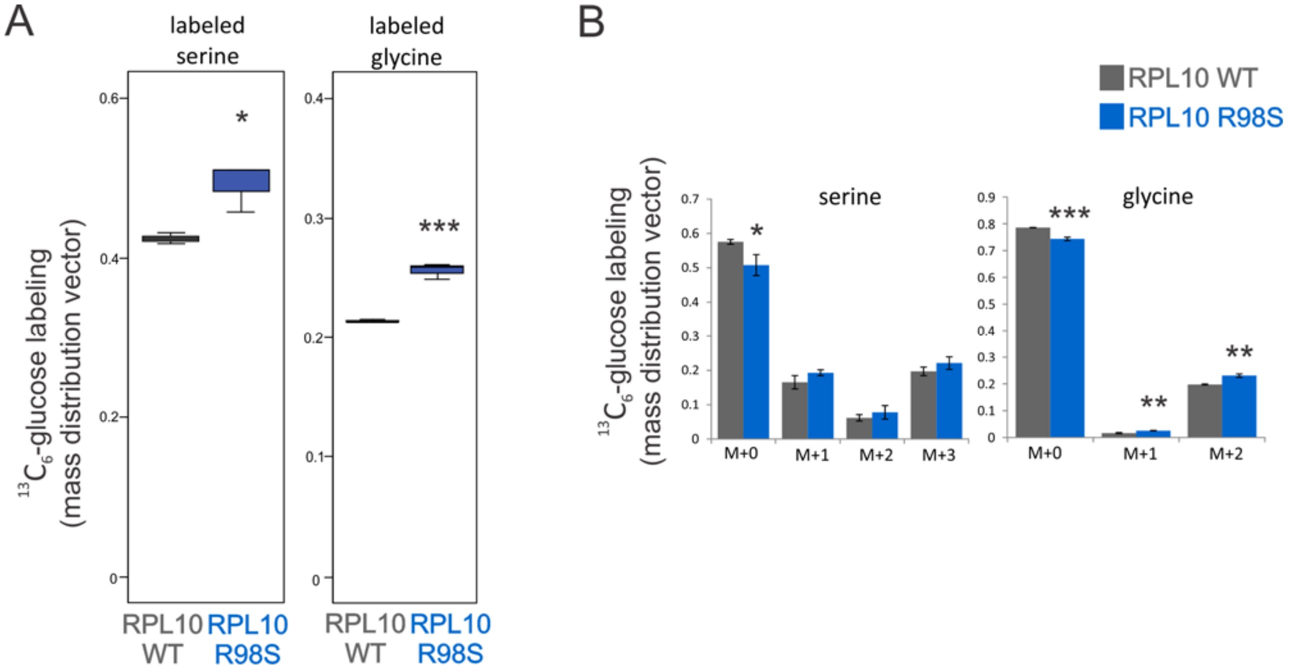

Supplementary Figure 9. Serine/glycine synthesis upregulation in *RPL10 R98S* Jurkat T-ALL cells.

Metabolic tracer analysis using $^{13}C_6$-Glucose, measuring the enrichment of serine and glycine from labeled glucose. Technical triplicates for *RPL10 WT* (grey) Jurkat and *RPL10 R98S* (blue) cells are presented. **A.** Overall labeled serine and glycine levels derived from $^{13}C_6$-Glucose. All box-plots show the median and error bars define data distribution. **B.** Mass distribution vector of all individual carbon labeled groups of serine and glycine derived from $^{13}C_6$-Glucose. Data are presented as mean ± standard deviation. Individual datapoints are shown. Statistical analysis * p-value< 0.05, ** p-value < 0.01, *** p-value < 0.001. P-values were calculated using a two-tailed student's t-test.

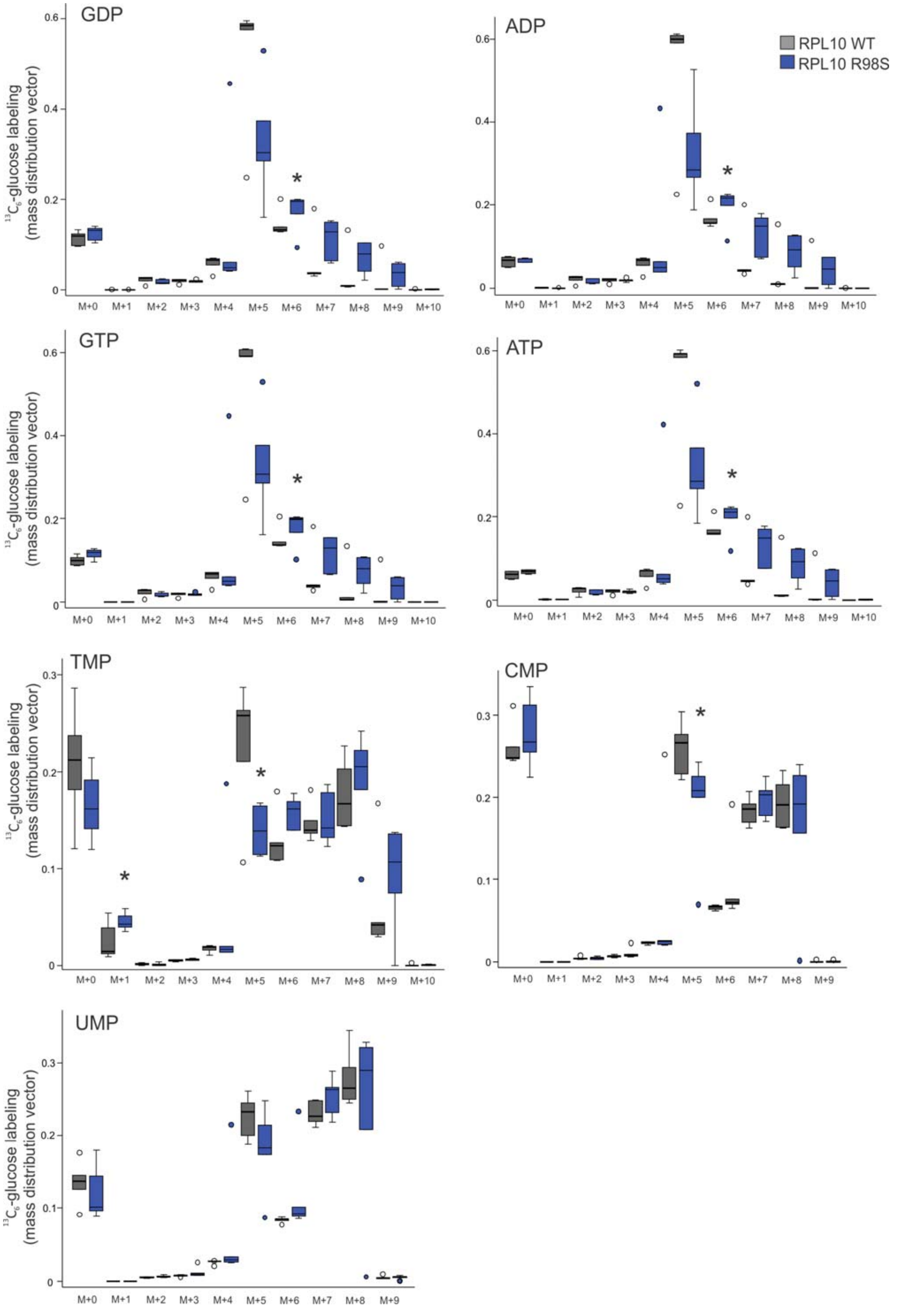
GDP
ADP
GTP
ATP
TMP
CMP
UMP
RPL10 WT
RPL10 R98S
13C6-glucose labeling (mass distribution vector)
M+0
M+1
M+2
M+3
M+4
M+5
M+6
M+7
M+8
M+9
M+10
*

Supplementary Figure 10. $^{13}C_6$-Glucose metabolite tracing into purine and pyrimidine bases. Metabolic tracer analysis using $^{13}C_6$-Glucose, measuring serine/glycine and associated tracing into purines (GDP, ADP, GTP and ATP) and pyrimidines (TMP, CMP and UMP). In this experiment n=6 independent Ba/F3 *RPL10 WT* (grey) clones versus n=5 *RPL10 R98S* (blue) clones are analyzed. All box-plots show the median and error bars define data distribution. Statistical analysis * p-value< 0.05, ** p-value < 0.01, *** p-value < 0.001. P-values were calculated using a two-tailed student's t-test.

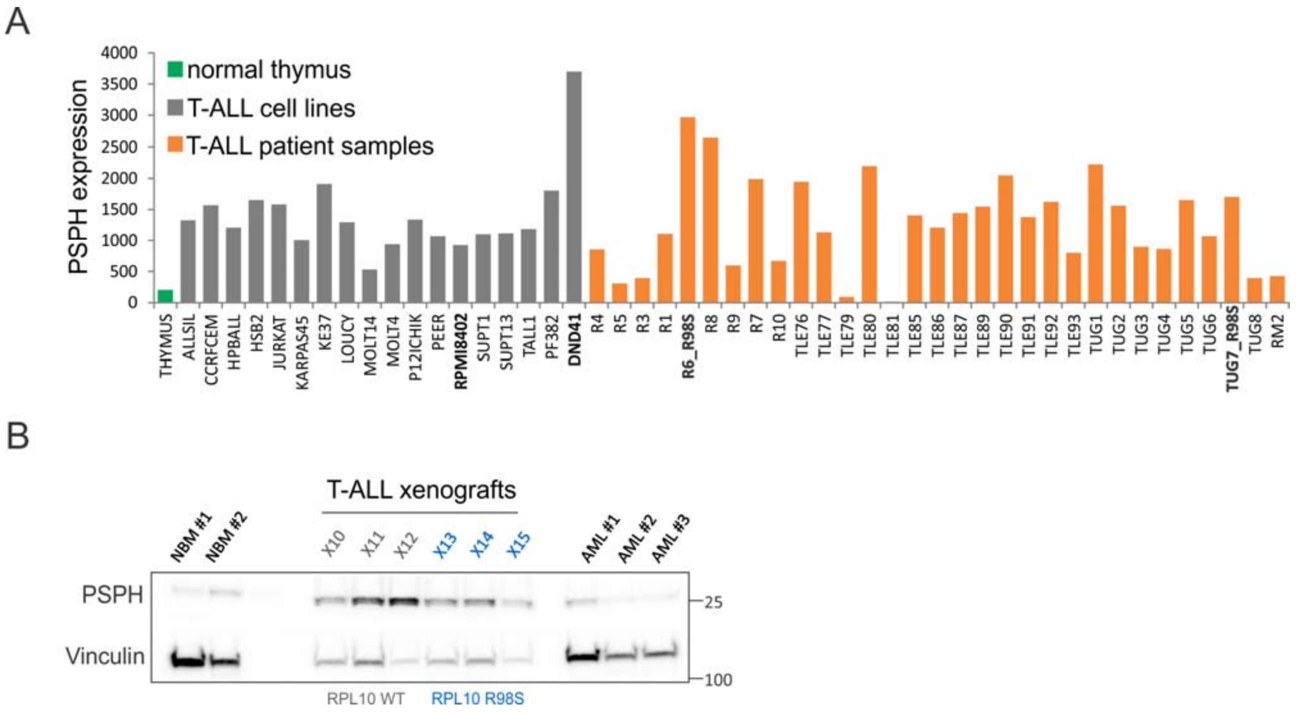

Supplementary Figure 11. PSPH expression in T-ALL cell lines and patient samples.

A. PSPH mRNA expression levels from mRNA sequencing data generated by Atak et al.[2] in normal thymus (red bar), T-ALL cell lines (grey bars) and T-ALL patient samples (orange bars). Of the T-ALL patient samples, the two *RPL10 R98S* positive samples are indicated as R6_R98S and TUG7_R98S. **B.** Immunoblot analysis of PSPH levels in normal bone marrow CD34 positive samples (NBM #1 and NBM #2), T-ALL xenografts (X10-X15) and AML samples (AML #1, #2 and #3). RPL10 WT cases are indicated in grey and R98S cases in blue. Vinculin served as loading control.

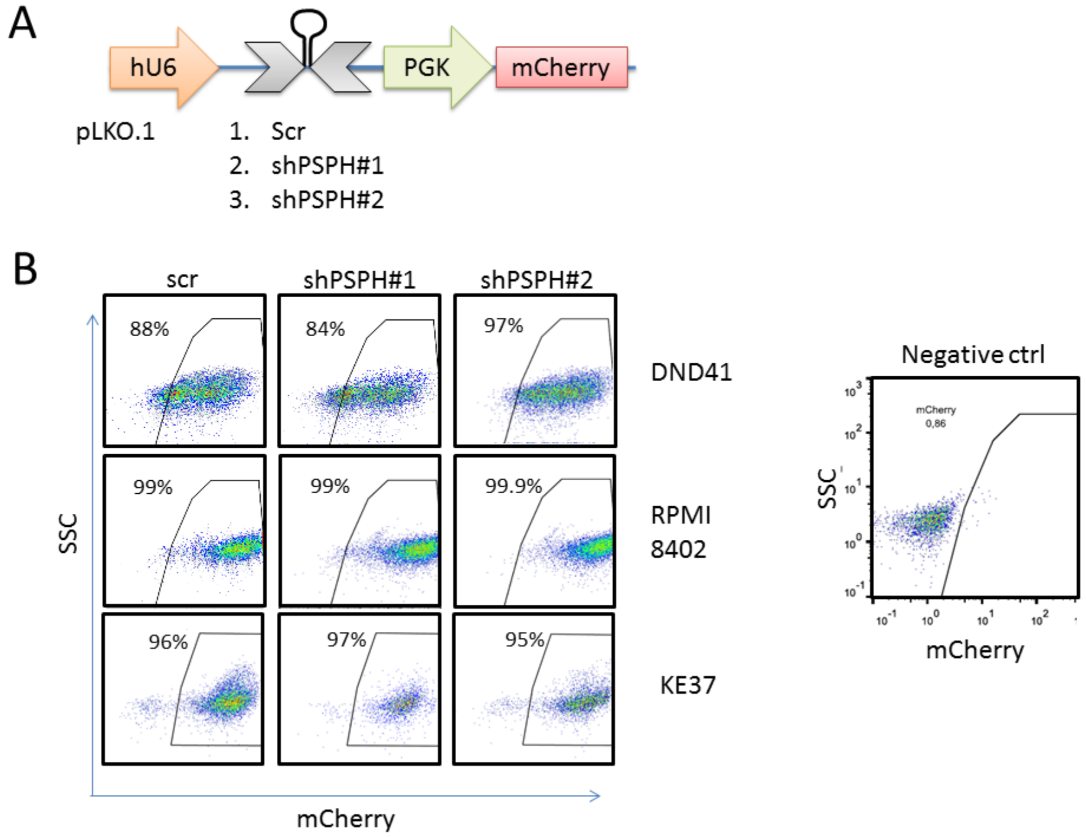

Supplementary Figure 12. PSPH shRNA vectors and transduction efficiencies.

A. shRNA hairpins that target PSPH or a scrambled control were cloned into the human U6 promoter-driven pLKO.1 vector containing a mCherry fluorescent protein coding sequence. **B.** Transduction efficiencies of T-ALL cell lines DND41, RPMI8402 and KE37 as assessed by flow cytometry analysis of mCherry expressing cells for the scrambled control, shPSPH#1 and shPSPH#2. Untranduced cells are used as negative ctrl for gate setting. The gating strategy shown in this panel was used for the analyses shown in Supplementary Figure 13.

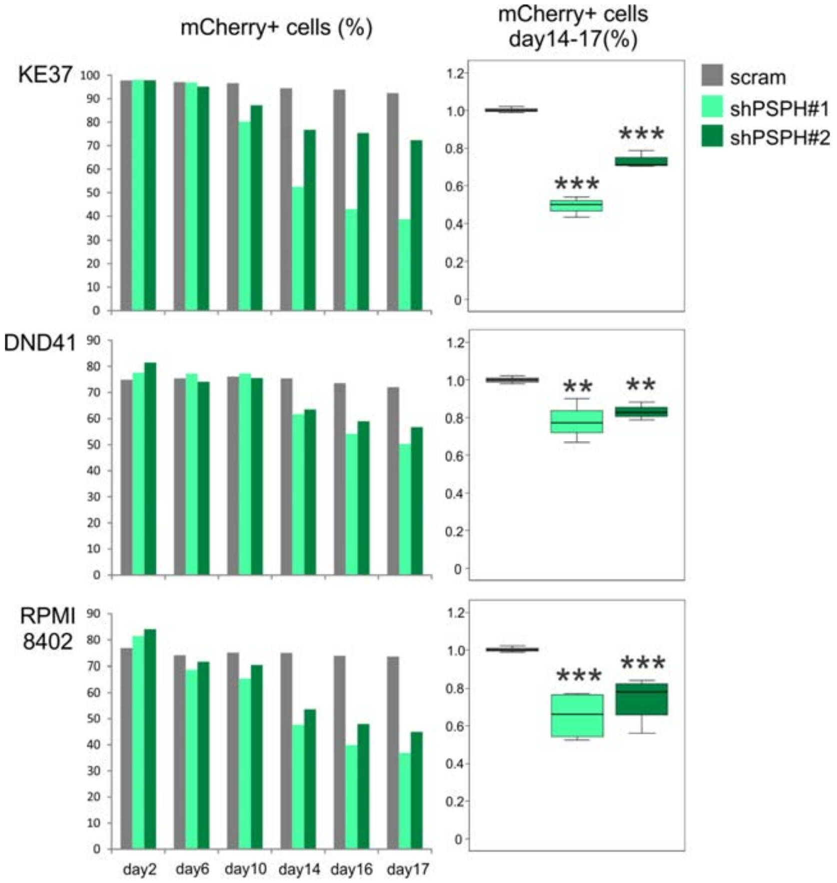

Supplementary Figure 13. Monitoring of mCherry labeled cells containing a PSPH targeting shRNA

Left panels represent the evolution of the % mCherry positive cells containing an shRNA over time, comparing scrambled control (grey) with shPSPH#1 (light green) and shPSPH#2 (dark green) in three independent T-ALL cells lines (upper: KE37; middle: DND41; lower: RPMI8402. Right panels show the quantification of the loss of mCherry expressing shPSPH cells as compared to the scrambled control. Box-plots show the median and error bars define data distribution. Statistical analysis ** p-value < 0.01, *** p-value < 0.001. P-values were calculated using a two-tailed student's t-test.

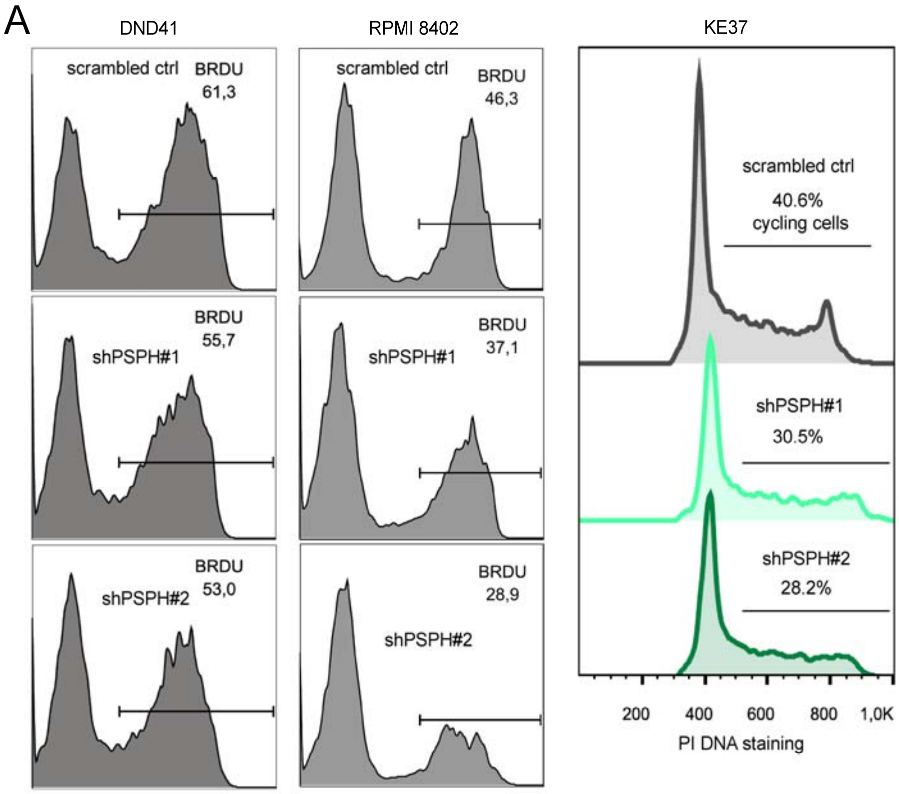

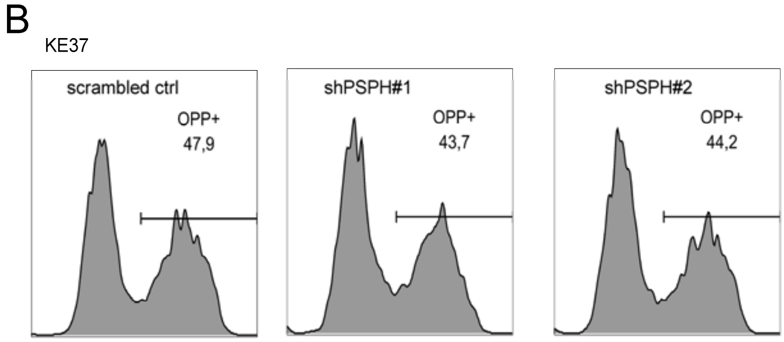

Supplementary Figure 14. Gating settings for BRDU and PI cell cycle analysis of PSPH knockdown cells. A. Gating settings for flow cytometry analysis of PSPH knockdown T-ALL cells measuring BRDU incorporation in cycling cells and PI DNA staining. These gating settings correspond to Figure 6D. **B.** Gating settings for flow cytometry OPP incorporation protein synthesis analysis in scrambled controls and PSPH knockdown cells. These gating settings correspond to Figure 6F.

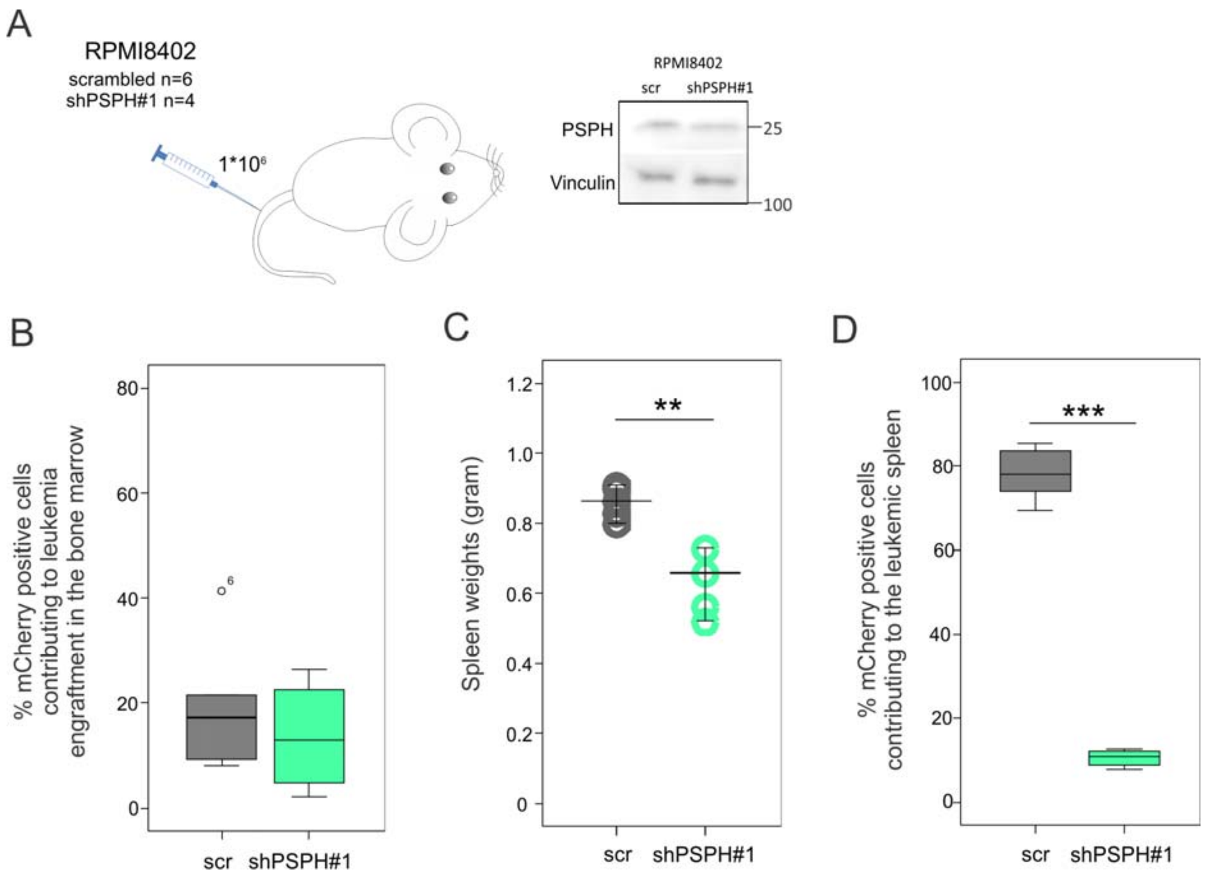

Supplementary Figure 15. PSPH knockdown reduces *in vivo* leukemia expansion potential of RPMI8402 T-ALL cells.

A. Left: schematic set-up to test the effect of PSPH knockdown on RPMI8402 leukemia progression *in vivo* in mice. Right: immunoblot analysis of PSPH protein expression levels in the RPMI8402 cells that were injected into NSG recipient mice. **B.** The percentage of mCherry expressing cells detected in the bone marrow of RPMI8402 injected mice. **C.** Reduced leukemia cell invasion in the spleen resulting in decreased spleen weights of mice injected with shPSPH#1 (light green) RPMI8402 leukemic cells as compared to scrambled control (grey) cell injected mice. **D.** The percentage of mCherry expressing scrambled control or shPSPH#1 leukemic cells that contributed to the enlarged spleen of RPMI8402 xenografted mice. Box-plots show the median and error bars define data distribution. Statistical analysis ** p-value < 0.01, *** p-value < 0.001. P-values were calculated using a two-tailed student's t-test.

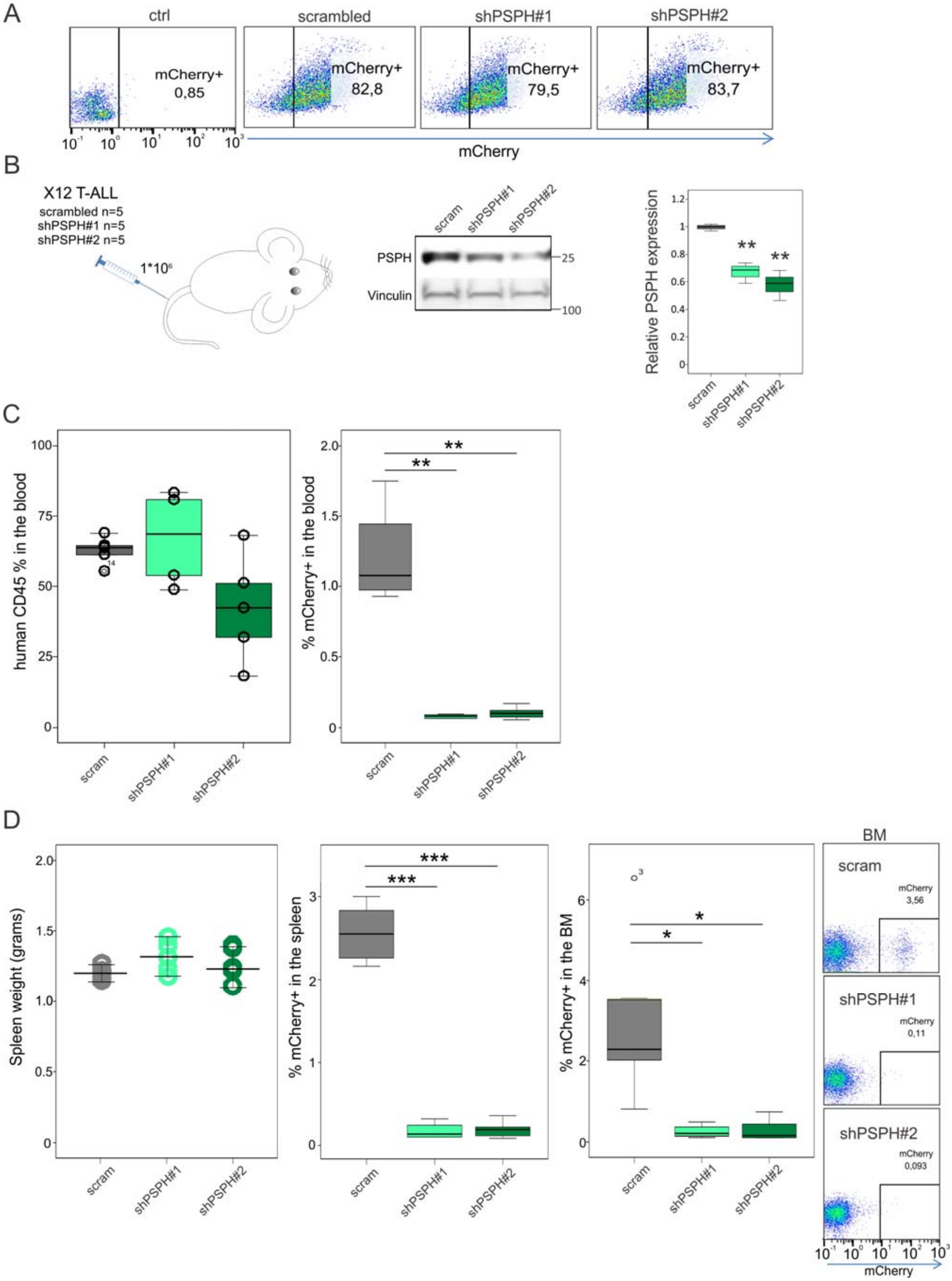

A
ctrl
scrambled
shPSPH#1
shPSPH#2
mCherry+
0,85
82,8
79,5
83,7
mCherry
B
X12 T-ALL
scrambled n=5
shPSPH#1 n=5
shPSPH#2 n=5
1*10^6
scram
PSPH
Vinculin
25
100
Relative PSPH expression
C
human CD45 % in the blood
% mCherry+ in the blood
D
Spleen weight (grams)
% mCherry+ in the spleen
% mCherry+ in the BM
BM
scram
mCherry
3,56
0,11
0,093

Supplementary Figure 16. PSPH knockdown suppresses *in vivo* leukemia expansion of X12 PDX T-ALL.

A. Flow cytometry analysis of transduction efficiencies of X12 PDX cells. Untranduced X12 cells served as a negative control for gate setting. Transduced cells are mCherry positive. **B.** Left: scheme of experimental design to test the effect of PSPH knockdown on X12 PDX T-ALL leukemia progression *in vivo* in mice. Middle: immunoblot analysis of PSPH protein expression levels in the X12 PDX T-ALL cells that were injected into NSG recipient mice. Right: quantification of immunoblots shown in the middle. **C.** Left: percentages of human CD45 X12 PDX T-ALL expressing cells in the blood of injected NSG mice two months after tail vein injection. Right: percentage of mCherry expressing cells detected in the peripheral blood of mice transplanted with X12 PDX T-ALL cells. **D.** Left: spleen weights of mice injected with X12 PDX T-ALL cells. Middle: The percentage of mCherry expressing cells detected in the spleens and bone marrow (BM) of mice transplanted with X12 PDX T-ALL shPSPH#1 (light green) and shPSPH#2 (dark green) leukemic cells as compared to scrambled control (grey) cell injected mice. Right: reduced leukemia cell invasion in the bone marrow (BM) derived from X12 PDX T-ALL shPSPH#1 and shPSPH#2 leukemic cells as compared to scrambled control cells injected in mice shown by flow cytometric dot-plot analysis. Box-plots show the median and error bars define data distribution. Statistical analysis * p-value < 0.05, ** p-value < 0.01, *** p-value < 0.001. P-values were calculated using a two-tailed student's t-test.

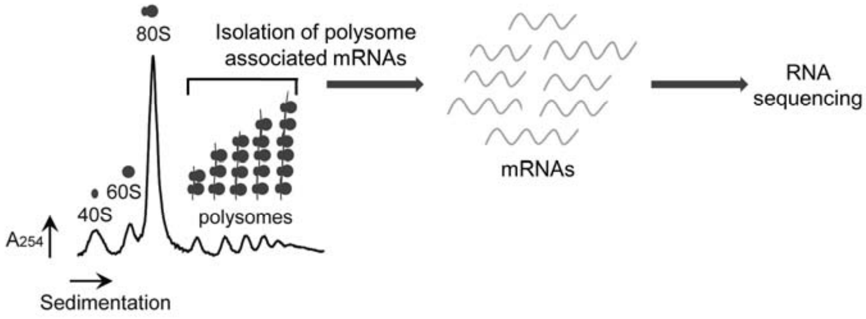

Supplementary Figure 17. Polysome profiling and RNA sequencing of polysome associated mRNA
Cell lysates were applied on sucrose gradients to obtain polysomes. mRNAs associated to polysomal fractions were subsequently isolated and sequenced.

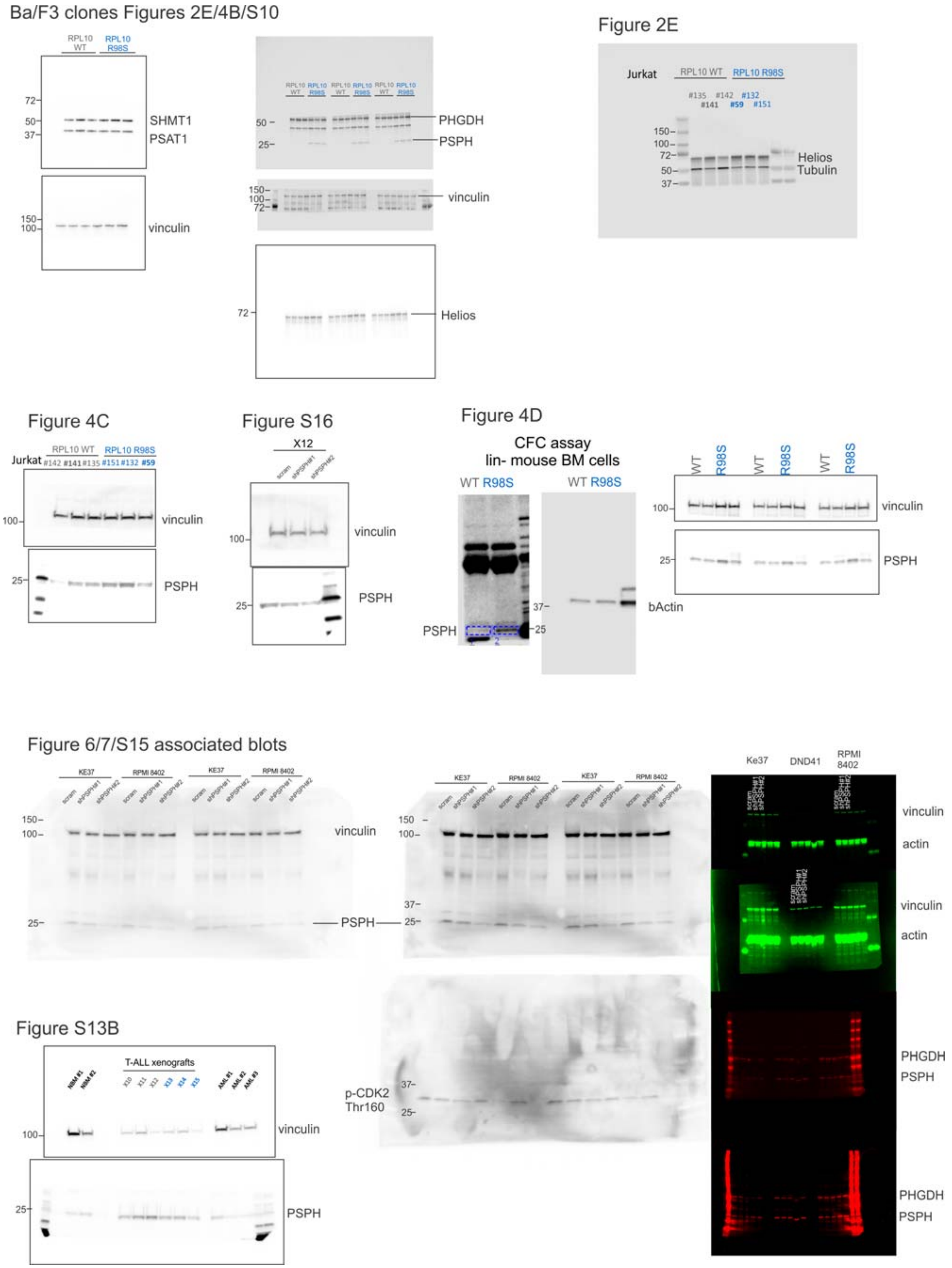

Supplementary Figure 18. Full immunoblots as presented in the corresponding figures.

SUPPLEMENTARY REFERENCES

1.Wang, J., Duncan, D., Shi, Z. & Zhang, B. WEB-based GEne SeT AnaLysis Toolkit (WebGestalt): update 2013. *Nucleic Acids Res* **41,** W77–83 (2013).

2.Atak, Z. K. et al. Comprehensive analysis of transcriptome variation uncovers known and novel driver events in T-cell acute lymphoblastic leukemia. *PLoS Genet* **9,** e1003997 (2013).
