# Supplementary material for: Translatome analysis reveals altered serine and glycine metabolism in T-cell acute lymphoblastic leukemia cells
Source: Nat Commun. 2019 Jun 11;10:2542. doi: 10.1038/s41467-019-10508-2 (PMC6559966; doi:10.1038/s41467-019-10508-2)
Supplement: Supplementary file 3 — Description of Additional Supplementary Files [file 41467_2019_10508_MOESM3_ESM.pdf]

Description of Additional Supplementary Files

File Name: Supplementary Data 1
Description: Genes with significant transcriptional up- or and downregulation in RPL10 R98S Ba/F3 cells

File Name: Supplementary Data 2
Description: Enrichment analyses using WebGestalt

File Name: Supplementary Data 3
Description: iRegulon prediction of transcriptional master regulators of upregulated mRNAs in RPL10 R98S cells

File Name: Supplementary Data 4
Description: iRegulon prediction of transcriptional master regulators of downregulated mRNAs in RPL10 R98S cells

File Name: Supplementary Data 5
Description: Genes with different translational efficiency between RPL10 R98S and RPL10 WT based on ribosome footprinting

File Name: Supplementary Data 6
Description: Genes with different translational efficiency between RPL10 R98S and RPL10 WT based on polysomal RNA sequencing

File Name: Supplementary Data 7
Description: Differentially expressed proteins between RPL10 R98S and WT

File Name: Supplementary Data 8
Description: Analysis of serine/glycine synthesis genes/proteins

File Name: Supplementary Data 9
Description: Biotinylated rRNA depletion oligos

File Name: Supplementary Data 10
Description: Primer sequences

File Name: Supplementary Data 11
Description: Datasets used in this study
