# Supplementary material for: Translatome analysis reveals altered serine and glycine metabolism in T-cell acute lymphoblastic leukemia cells
Source: Nat Commun. 2019 Jun 11;10:2542. doi: 10.1038/s41467-019-10508-2 (PMC6559966; doi:10.1038/s41467-019-10508-2)
Supplement: Supplementary file 15 — Reporting Summary [file 41467_2019_10508_MOESM15_ESM.pdf]

nature research

Corresponding author(s): Kim De Keersmaecker

Reporting Summary

Nature Research wishes to improve the reproducibility of the work that we publish. This form provides structure for consistency and transparency in reporting. For further information on Nature Research policies, see Authors & Referees and the Editorial Policy Checklist.

Statistical parameters

When statistical analyses are reported, confirm that the following items are present in the relevant location (e.g. figure legend, table legend, main text, or Methods section).

n/a	Confirmed	
☐	☒	The exact sample size (*n*) for each experimental group/condition, given as a discrete number and unit of measurement
☐	☒	An indication of whether measurements were taken from distinct samples or whether the same sample was measured repeatedly
☐	☒	The statistical test(s) used AND whether they are one- or two-sided<br>*Only common tests should be described solely by name; describe more complex techniques in the Methods section.*
☒	☐	A description of all covariates tested
☐	☒	A description of any assumptions or corrections, such as tests of normality and adjustment for multiple comparisons
☐	☒	A full description of the statistics including central tendency (e.g. means) or other basic estimates (e.g. regression coefficient) AND variation (e.g. standard deviation) or associated estimates of uncertainty (e.g. confidence intervals)
☐	☒	For null hypothesis testing, the test statistic (e.g. *F*, *t*, *r*) with confidence intervals, effect sizes, degrees of freedom and *P* value noted<br>*Give P values as exact values whenever suitable.*
☒	☐	For Bayesian analysis, information on the choice of priors and Markov chain Monte Carlo settings
☒	☐	For hierarchical and complex designs, identification of the appropriate level for tests and full reporting of outcomes
☐	☒	Estimates of effect sizes (e.g. Cohen's *d*, Pearson's *r*), indicating how they were calculated
☐	☒	Clearly defined error bars<br>*State explicitly what error bars represent (e.g. SD, SE, CI)*

Our web collection on statistics for biologists may be useful.

Software and code

Policy information about availability of computer code

Data collection	Not applicable
Data analysis	All statistics were performed using the R software (and the following R packages: Babel, DESeQ2) or IBM SPSS 23 (IBM Analytics) software. The following open source softwares were also used for analysis: seqtk, fastq-mcf, Bowtie, TopHat2, iRegulon, WebGestalt.<br>All used softwares are referenced in the text.<br>Custom code was used for metagene analyses around start and stop codons and ribosome footprint density profiles as described in the methods section.

For manuscripts utilizing custom algorithms or software that are central to the research but not yet described in published literature, software must be made available to editors/reviewers upon request. We strongly encourage code deposition in a community repository (e.g. GitHub). See the Nature Research guidelines for submitting code & software for further information.

Data

Policy information about availability of data

All manuscripts must include a data availability statement. This statement should provide the following information, where applicable:

- Accession codes, unique identifiers, or web links for publicly available datasets
- A list of figures that have associated raw data
- A description of any restrictions on data availability

All RNA sequencing and proteomics datasets were generated from isogenic mouse lymphoid Ba/F3 cells engineered to express the WT or R98S mutant form of human RPL10, with three independent WT and three R98S cell clones analyzed in each experiment. The quantitative mass spectrometry dataset has been described previously (PRIDE identifier PXD005995)(Girardi et al., Leukemia, 2018), as well as polysomal RNA sequencing and its associated mRNA sequencing (GEO accession GSE106528) (Girardi et al., Leukemia, 2018) and a supplementary mRNA sequencing dataset (GEO accession GSE106530)(Kampen et al, Leukemia, 2018). Ribosome footprinting and matched mRNA sequencing (GEO accession GSE106529) have not been described previously. All datasets are listed in Supplementary Table 11. These datasets are linked to Figures 1, 2 and 3 and Supplementary Figures 1-4. There are no restrictions on data availability.

Field-specific reporting

Please select the best fit for your research. If you are not sure, read the appropriate sections before making your selection.

☒ Life sciences ☐ Behavioural & social sciences ☐ Ecological, evolutionary & environmental sciences

For a reference copy of the document with all sections, see nature.com/authors/policies/ReportingSummary-flat.pdf

Life sciences study design

All studies must disclose on these points even when the disclosure is negative.

Sample size	No sample-size calculations were performed. Sample sizes were chosen to have enough replicates to do statistics and were determined by availability of biological samples and/or based on experience with our biological models.
Data exclusions	total RNA seq sample 1-WT36 matched with the polysome profiling was excluded because it clustered with the R98S mutants in PCA analysis. Besides this, no data were excluded.
Replication	All figure legends of experimental data contain clear descriptions of the biological and technical replicates.
Randomization	Allocation of mice to experimental or control groups was random.
Blinding	Blinding was not performed.

Reporting for specific materials, systems and methods

Materials & experimental systems

n/a	Involved in the study	
☐	☒	Unique biological materials
☐	☒	Antibodies
☐	☒	Eukaryotic cell lines
☒	☐	Palaeontology
☐	☒	Animals and other organisms
☐	☒	Human research participants

Methods

n/a	Involved in the study	
☒	☐	ChIP-seq
☐	☒	Flow cytometry
☒	☐	MRI-based neuroimaging

Unique biological materials

Policy information about availability of materials

Obtaining unique materials	RPL10 WT and RPL10 R98S Ba/F3 and Jurkat cell models are available on request upon completion of an MTA. Shipping costs will be paid by the requesting researcher.

Antibodies

Antibodies used	Western blotting:<br>PSPH (Proteintech, 14513-1-AP), phospho-CDK2 or Helios (Cell Signaling Technology, #2561 and #42427), and with secondary Goat Anti-Rabbit or Goat Anti-Mouse IgG-HRP antibody (ThermoFischer Scientific, 31432 and 31462) or dylight anti-Mouse or anti-Rabbit antibodies (Cell Signaling Technology # 5470 and # 5151). Quantification was performed using LI-COR Image Studio Lite software version 5.2. Vinculin (Sigma Aldrich, V9131) or β-actin (Sigma Aldrich, A1978).<br>Flow cytometry:<br>for the PDX xenograft experiments an anti-human CD45 antibody was used (BD, 557659). Anti-BRDU antibody (Thermo Fisher, clone BU20A, 11-5071-42) was used to stain BRDU incorporated into the DNA of cycling cells.
Validation	Western blotting: All antibodies used showed a specific band at the expected MW according to the Precision Plus Protein Dual Color (Biorad) protein marker.<br>For flow cytometry: The anti-human CD45 antibody specificity was validated using counter staining with anti-mouse CD45. For BRDU flow cytometry, non-cycling cells were used as a negative control for aspecific BRDU incorporation and antibody staining.

Eukaryotic cell lines

Policy information about cell lines

Cell line source(s)	The Ba/F3 cell model and hematopoietic cell cultures derived from RPL10 R98S conditional knock-in mice were previously described (Girardi et al., Leukemia, 2018) . Jurkat, DND41, RPMI8402 and KE37 T-ALL cell lines were obtained from Leibniz-Institute DSMZ.
Authentication	RPMI8402 and DND41 were authenticated by confirming unique NOTCH1 mutational status. Other used cell lines were not authenticated.
Mycoplasma contamination	Cell lines were tested for mycoplasma on a regular basis and always tested negative.
Commonly misidentified lines (See ICLAC register)	*Name any commonly misidentified cell lines used in the study and provide a rationale for their use.*

Animals and other organisms

Policy information about studies involving animals; ARRIVE guidelines recommended for reporting animal research

Laboratory animals	Xenografting of human T-ALL samples was performed in 6-8 weeks old male or female NOD.Cg-Prkdcscid Il2rgtm1Wjl/SzJ (NSG) mice.
Wild animals	Not applicable
Field-collected samples	Not applicable

Human research participants

Policy information about studies involving human research participants

Population characteristics	For the experiments using human T-ALL xenograft material, approval was obtained by the ethics committees of UZ/ KU Leuven and Universiteit Gent (S54608 and S59975). After getting written informed consent, the mononuclear cell fraction of bone marrow from pediatric T-ALL patients was obtained and the cells were xenografted into NSG mice.
Recruitment	Human T-ALL samples from patients that gave approval have been systematically collected by our collaborator Jan Cools and have been injected into NSG mice for expansion.

Flow Cytometry

Plots

Confirm that:

- [x] The axis labels state the marker and fluorochrome used (e.g. CD4-FITC).
- [x] The axis scales are clearly visible. Include numbers along axes only for bottom left plot of group (a 'group' is an analysis of identical markers).
- [x] All plots are contour plots with outliers or pseudocolor plots.
- [x] A numerical value for number of cells or percentage (with statistics) is provided.

Methodology

Sample preparation	All measurements were performed on suspension hematopietic cell lines. Proliferation was measured by counting viable cells

Sample preparation	over time (growth curve), and cell cycle was analyzed by Bromodeoxyuridine (BrdU) incorporation and/or propodium iodide (PI) staining. BrdU incorporation was measured following 70% EtOH fixation, HCl denaturation, EDTA neutralization and BrdU-FITC antibody staining (Thermo Fisher). PI cell cycle analysis required 70% EtOH fixation followed by 1 hour incubation with PI solution (25 μg/mL PI, 300 μg/mL RNAse, 0.05% Triton-X100, Sigma Aldrich) at 37°C. Protein translation was assessed using O-propargyl-puromycin (OPP) incorporation according to manufacturer's protocol with the adaptation of methanol fixation (Click-iT, Thermo Fisher).
Instrument	All samples were measured using a MACSQuant VYB (Miltenyi) flow cytometer
Software	FlowJo software was used for all data analyses
Cell population abundance	No cell sortings were done
Gating strategy	Gating settings for the FACS analyses are shown in Supplementary Figures 12, 14 and 16. For Supplementary Figure 16, this is in-figure, for Supplementary Figure 12 and 14, we refer to the corresponding figure where this gating strategy was used in the supplementary figure legend. Gating was performed based on unstained/untransduced cells.

☒ Tick this box to confirm that a figure exemplifying the gating strategy is provided in the Supplementary Information.
